# Short-Term Memory Impairment and Left Dorsolateral Prefrontal Cortex Dysfunction in the Orthostatic Position: A Single Case Study of Sinking Skin Flap Syndrome

**DOI:** 10.1155/2015/318917

**Published:** 2015-10-27

**Authors:** Luca Sebastianelli, Verena Stoll, Viviana Versace, Sara Martignago, Stephan Obletter, Marco Lavoriero, Kathrin Malfertheiner, Gertraud Gisser, Leopold Saltuari

**Affiliations:** ^1^Krankenhaus Sterzing, Abteilung Neurorehabilitation, Sterzing, 39049 Bozen, Italy; ^2^Research Unit for Neurorehabilitation of South Tyrol, 39100 Bozen, Italy; ^3^Hochzirl Hospital, Department of Neurology, 6170 Zirl, Austria

## Abstract

We describe the case of a patient who underwent craniectomy for hemorrhage of the left parietal lobe. Three weeks later, orthostatic memory impairment was detected as initial symptom of sinking skin flap syndrome (SSFS). This deficit was examined by neuropsychological testing and associated with a posture-dependent increase in the delta/alpha ratio at the F3 electrode, an electroencephalographic (EEG) index related to brain hypoperfusion. This EEG spectral alteration was detected in a brain region that includes the left dorsolateral prefrontal cortex, an area known to be involved in memory processing; therefore we hypothesize that SSFS induced reversible hypoperfusion of this otherwise undamaged cortical region. Neither of these findings was present after cranioplasty. This case suggests that SSFS may induce neuropsychological deficits potentially influencing outcome in the postacute phase and is further evidence supporting the clinical benefits of early cranioplasty.

## 1. Introduction

Decompressive craniectomy may have several complications. Among these, sinking skin flap syndrome (SSFS) has been defined as the onset of diverse neurological deficits that occurs progressively while the scalp flap sinks into the skull defect. Skin displacement is a consequence of the gradient between intracranial and atmospheric pressure and leads to dysfunction in the compressed cerebral structures, by inducing region-specific perfusion deficit. Hence, symptoms and severity of SSFS are related to function of the involved brain areas, and both motor and cognitive deficits are reported [1, 2]. In particular, disturbances in memory, executive, and planning functions are described in patients who underwent frontal craniectomy [3]. Other factors, such as disturbances in cerebrospinal fluid (CSF) circulation, and generalized dysregulation of cerebral blood flow may also play a role in nonfocal neurological deficits, as described in the “syndrome of the trephined,” which includes headache, dizziness, alterations in behaviour and mood, seizures, and fatigue [4]. “Paradoxical herniation” is the most advanced stage of the syndrome. This potentially life-threatening clinical picture occurs when the sinking flap induces displacement of brain midline structures.

Early symptoms can be ameliorated by horizontal recumbent position, which increases intracranial pressure. However, only cranial vault reconstruction can reverse the syndrome by restoring physiological mechanisms that control intracranial pressure [5].

EEG activity is significantly influenced by cognitive and memory performance, and specific event-related changes in oscillation frequency, occurring in definite brain areas, have been associated with different cognitive tasks. For instance, theta activity increases in prefrontal cortex during successful working memory formation, whereas attentional demands lead to a selective reduction of specific alpha frequencies [6].

Furthermore, quantitative EEG (qEEG) is increasingly regarded as a valuable tool to study cerebral perfusion, since a direct relationship exists between cerebral blood flow reduction and early increase of low frequency band power [7]. Different qEEG indexes have been proposed in order to study cerebral perfusion in different clinical situations. Among these, ratio between power of alpha and delta band (DAR) has been proven to be an early index of brain hypoperfusion and to have better sensibility and specificity for brain hypoperfusion with respect to other qEEG indexes [8].

## 2. Case Presentation

A 63-year-old right-handed, trilingual (Ladin, German and Italian) man underwent frontotemporoparietal craniectomy after non-traumatic cerebral bleeding in the left parietal lobe. He had severe right-sided hemiparesis and expressive aphasia but was able to communicate with a reliable yes-no code. 14 days after the procedure, an initial depression in the anterior portion of the skin flap was detected, which reversed after clinostatic recumbency. Furthermore, a greater difficulty in following instructions was observed during physical therapy in standing position. This condition was hypothesized to be an initial symptom of SSFS.

In order to verify whether SSFS induced worse cognitive performance in upright position, we performed four assessments pairing neuropsychological tests to EEG recordings, in both horizontal and vertical positions, either before or after cranioplasty. “Clinostatic” position was defined as the patient lying down in bed after night rest, while “orthostatic” position was defined as the patient sitting for two hours.

As the patient had to be tested soon after injury and due to its preferred language for testing, we used a recently introduced German battery specifically designed for patients in the early stage of recovery after a stroke (Cologne Neuropsychological Screening for Stroke Patients; German: Kölner Neuropsychologisches Screening für Schlaganfall-Patienten (KöpSS)) [9].

One minute after the last neuropsychological test, 5 minutes of resting eye-closed state EEG was recorded using a standard 10–20 EEG-cap. Signals were acquired on a Nicolet EEG v32 (CareFusion Corp., San Diego, CA, USA), referenced to the earlobes (A1 + A2), and digitized at 500 Hz. Impedances were < 5 KΩ. Spectral data were calculated for each electrode by fast Fourier transform in the range of 0.5–30 Hz (Nicolet vEEG v5.71.6.2577). Frequency bands were defined as follows: delta (0.5–4.0 Hz); theta (4.0–8.0 Hz); alpha (8.0–13.0 Hz); beta (13.0–30.0 Hz). Since absolute power may be influenced by the cranial breach, flap movements, or later on the cranial vault reconstruction, we avoided using absolute band power in data analysis. Rather, we normalized spectral data in each electrode to its total cumulative power for each epoch [10]. Therefore, the power of each band is expressed as a rate, being total power = 1. For each of the four EEG recordings, 20 nonoverlapping epochs of 1 second were visually identified to be free of artefacts. A threshold of 40 *µ*V in the electrooculogram was used to reject sequences containing blinks or eye movements. For each of these epochs, normalized band powers were obtained and DAR was calculated for each electrode. The 20 DAR values were then mediated and are expressed in text as mean ± standard error of the mean. *t*-test was used to assess statistical significance of quantitative EEG results.

Results of neuropsychological assessments are summarized in Table 1. Before cranioplasty, the patient was only able to perform some KöpSS tests. Among them, drawing and selective attention tasks were not affected by posture. He was not able to perform the mental rotation and working memory tasks, as he did not understand the instructions in the specified timeframe. Two tests assessed short-term memory, and he obtained worse scores in the orthostatic position compared with clinostatism. In particular, for the second test, the score decreased from 9/10 to 6/10. After cranioplasty, he showed improvements in several tests but with no differences between the two postures.

We hypothesized that this cognitive deficit was due to hypoperfusion of left cortex, compressed by the sinking skin flap. Therefore, we calculated DAR for each of the four conditions.

**Figure 1:**
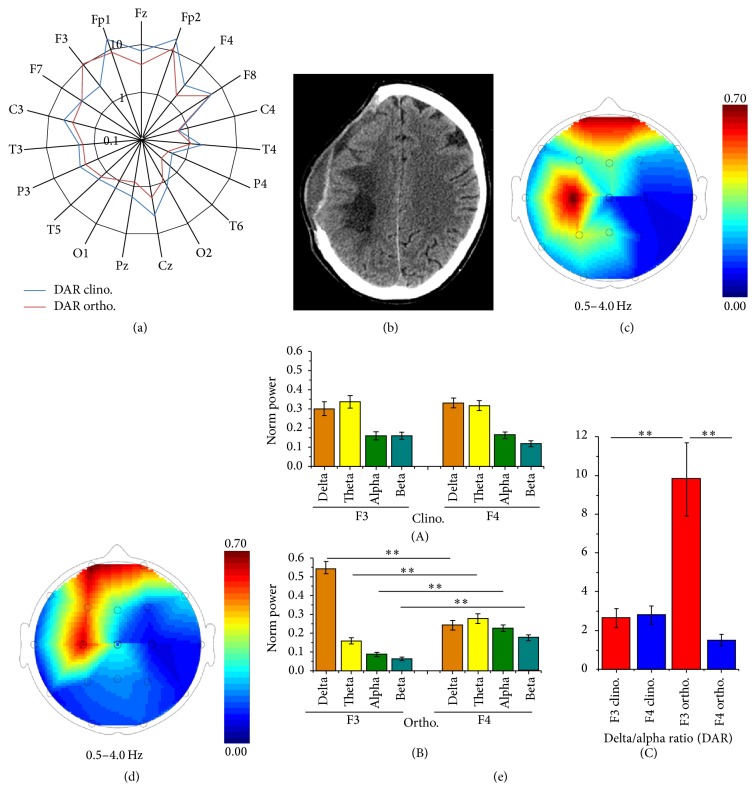
Precranioplasty findings. DAR value for each electrode in clino- and orthostatic positions, shown on a logarithmic scale (a). Brain CT scan showing the posthemorrhagic lesion and extent of craniotomy (b). Delta power band distribution maps in clino- (c) and orthostatic (d) positions. Mean normalized band powers of F3 and F4 signals in clino- ((e)(A)) and orthostatic ((e)(B)) positions. DAR in F3 and F4 in both positions ((e)(C)). *∗∗* indicates *p* < 0.001.

Before cranioplasty, a change in DAR value across the postural change was observed (Figure 1(a)). It decreased (*p* < 0.05) in frontal (Fp1, F4), midline (Fz, Cz, and Pz), and occipital (O1, O2) electrodes, while it increased (*p* < 0.001) in F3 electrode. Due to the extent of the craniotomy (Figure 1(b)), several electrodes were on the breach (F3, C3, P3, F7, and T3), but a DAR increase was observed only in F3. Accordingly, in clinostatic position, the highest values of delta band power were recorded in correspondence of posthemorrhagic lesion and in frontal regions (Figure 1(c)), while in orthostatism this distribution extended to involve F3 region (Figure 1(d)). These maps are obtained from sample epochs of one second each, by normalizing spectral signals as described above. Therefore, a delta power value of 0.55 in F3 means that delta band is over the 50% of the total signals recorded by this electrode.

Figure 1(e) shows mean normalized band powers of F3 and F4: in clinostatism (Figure 1(e)(A)) band powers were similar for each homologue band, while, after 2 hours in the orthostatic position, an asymmetry was evident between these electrodes (Figure 1(e)(B)). In clinostatism, DAR was 2.66 ± 0.49 at F3 and 2.81 ± 0.46 at F4 (*p* > 0.05), while, in the orthostatic position, it was 9.81 ± 1.87 at F3 and 1.51 ± 0.29 at F4 (*p* < 0.001) (Figure 1(e)(C)).

**Figure 2:**
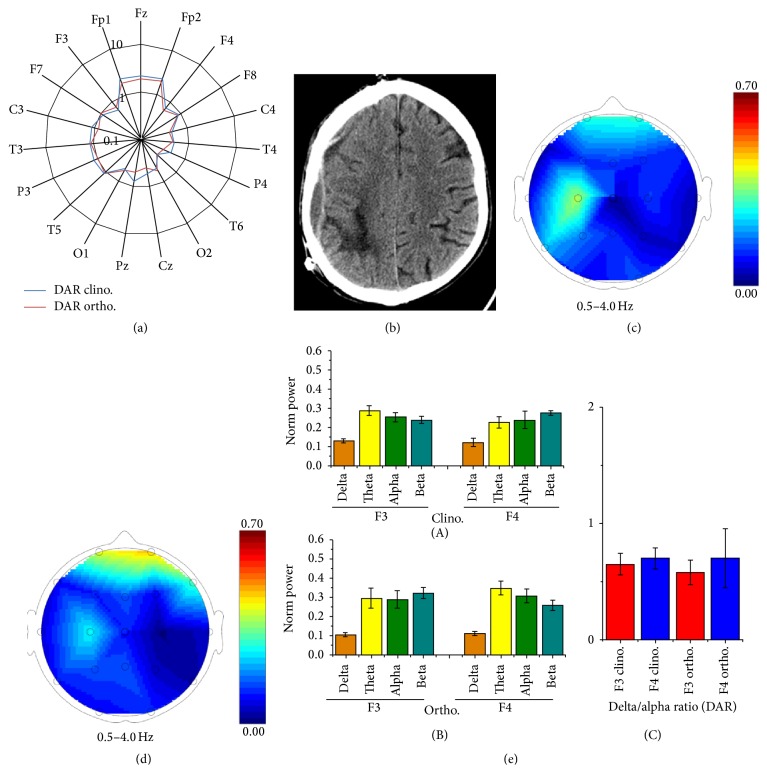
Postcranioplasty findings. DAR value for each electrode in clino- and orthostatic positions, shown on a logarithmic scale (a). Brain CT scan showing the posthemorrhagic lesion and cranioplasty results (b). Delta power band distribution maps in clino- (c) and orthostatic (d) positions. Mean normalized band powers of F3 and F4 signals in clino- ((e)(A)) and orthostatic ((e)(B)) positions. DAR in F3 and F4 in both positions ((e)(C)).

After cranioplasty, no posture-related DAR differences at any electrodes were observed (Figure 2(a), *p* > 0.05). Compared with the preoperative period, decreased DAR was observed diffusely. The spectral signals at F3 and F4 remained symmetrical (*p* > 0.05, Figures 2(c), 2(d), and 2(e)).

## 3. Discussion

In the case here reported, the patient exhibited a posture-related impairment in two tests assessing short-term memory. In these two tests, the patient had to observe 10 figures and after two specified time latencies recognize them among 10 distractors. The ability to execute such a visual old/new recognition task is related to correct functioning of the left dorsolateral prefrontal cortex (DLPFC) [11], an area known to be involved in visual short-term memory (STM) as well as in working memory and in mood control. Tasks that were not affected by postural change (letter cancelation and figure- and shape-copying tasks) assessed functions unrelated to the left DLPFC [12].

Quantitative analysis of EEG recordings before cranioplasty showed a significant increase in DAR in the region explored by the F3 electrode during orthostatism. According to the 10–20 system, correspondence between the F3 electrode and the left DLPFC (Brodmann's area 9/46) has been shown by comparing neuroimages and Talairach stereotactic coordinates [13] and by functional modulation [11, 14]. Interestingly, also event-related potential studies indicate the cortical region explored by the F3 electrode to be specifically involved in the familiarity effect during old/new recognition tasks [15].

DAR decrease observed in several electrodes across postural change was due to relative alpha and beta power increase and might be an effect of an excessive CSF scattering, which increases power of higher frequencies, as shown in healthy subjects [16]. Alternatively, it could be a consequence of the “breach effect,” which preferentially increases transmission of higher frequencies [17]. However, this possibility is unlikely, since DAR decreased also in the electrodes placed on the intact skull, and none of the features of “breach rhythm” were observed in the raw EEG. In any case, the opposite trend between F3 and the adjacent electrodes supports the specificity of DAR increase as index of hypoperfusion.

Postcranioplasty neuropsychological tests show improvement in several cognitive domains, which can be related to overall function in both hemispheres, without any posture-related change. Accordingly, EEG data were similar for clino- and orthostatic positions. Postcranioplasty distribution of delta power is more physiological, according to values reported for the eye-closed state of default mode network [18]. The possibility that bilateral functional improvement may be a consequence of cranioplasty cannot be ruled out.

Perfusion abnormalities in SSFS inducing focal dysfunction were previously described. We obtained similar results using methods that to the best of our knowledge are previously unreported for this clinical problem. Clinical usefulness of these tools should be therefore carefully evaluated in larger case series. However, we believe that such a correspondence between clinical and instrumental data observed in this case is not coincidental and deserves a detailed discussion.

Postacute management of craniectomy patients is a frequent issue in neurologic and neurosurgical clinical practice, since optimal timing and indications for cranioplasty are still a matter of debate. This case report suggests that SSFS may induce neuropsychological deficits by impairing function of an otherwise undamaged brain area.

New frontal dysfunctions that induce cognitive or mood disorders are difficult to unmask in patients with severe brain injury but may influence functional recovery. Onset of new motor deficits (a more typical sign of SSFS) may reflect an excessively advanced stage of the syndrome. Therefore, as it has been already suggested [19], early cranioplasty should be considered in these patients.

## Figures and Tables

**Table 1:** Results of neuropsychological assessment.

Domain and subtest (max score)	Clino. precranioplasty	Ortho. precranioplasty	Clino. postcranioplasty	Ortho. postcranioplasty
Orientation				
Person, space, and time (3)	0	0	0	0
Language				
Name (6)	0	0	0	0
Read (6)	0	0	0	0
Write (6)	0	0	0	0
Verbal comprehension (3)	2	2	3	3
Language expression (6)	0	0	0	0
Praxis				
Single actions (8)	0	0	6	5
Sequence of actions (10)	0	0	0	0
Visuospatial performance				
Perception of face expression (4)	0	0	0	0
Draw (6)	5	5	5	6
Mental rotation (3)	0	0	3	3
Count				
Mental count (7)	0	0	3	4
Executive performance and attention				
Selective attention (10)	2	3	4	5
Working memory (3)	0	0	2	2
Logic (1)	0	0	1	1
Short-term memory				
First latency (10)	6	4	7	7
Second latency (10)	9	6	7	8
